# Return to work after lumbar disc herniation surgery: an occupational cohort study

**DOI:** 10.1080/17453674.2021.1951010

**Published:** 2021-07-16

**Authors:** Raul Laasik, Petteri Lankinen, Mika Kivimäki, Marko H Neva, Ville Aalto, Tuula Oksanen, Jussi Vahtera, Keijo T Mäkelä

**Affiliations:** a Department of Orthopaedics and Trauma, Tampere University Hospital, Tampere, Finland;; b Department of Orthopedics and Traumatology, Turku University Hospital and University of Turku, Turku, Finland;; c Satakunta Central Hospital, Pori, Finland;; d Finnish Institute of Occupational Health, Helsinki, Finland;; e Clinicum, Faculty of Medicine, University of Helsinki, Helsinki, Finland;; f Department of Epidemiology and Public Health, University College London, London, UK;; g Institute of Public Health and Clinical Nutrition, University of Eastern Finland, Kuopio, Finland;; h Department of Public Health, University of Turku, and Centre for Population Health Research, University of Turku and Turku University Hospital, Turku, Finland

## Abstract

Background and purpose — Lumbar disc herniation is a common surgically treated condition in the working-age population. We assessed health-related risk factors for return to work (RTW) after excision of lumbar disc herniation. Previous studies on the subject have had partly contradictory findings.

Patients and methods — RTW of 389 (n = 111 male, n = 278 female; mean age 46 years, SD 8.9) employees who underwent excision of lumbar disc herniation was assessed based on the Finnish Public Sector Study (FPS). Baseline information on occupation, preceding health, and health-risk behaviors was derived from linkage to national health registers and FPS surveys before the operation. The likelihood of RTW was analyzed using Cox proportional hazard univariable and multivariable modelling.

Results — 95% of the patients had returned to work at 12 months after surgery, after on average 78 days of sickness absence. Faster RTW in the univariable Cox model was associated with a small number of sick leave days (< 30 days) before operation (HR 1.3, 95% CI 1.1–1.6); high occupational position (HR 1.6, CI 1.2–2.1); and age under 40 years (HR 1.5, CI 1.1–1.9). RTW was not associated with sex or the health-related risk factors obesity, physical inactivity, smoking, heavy alcohol consumption, poor self-rated health, psychological distress, comorbid conditions, or purchases of pain or antidepressant medications in either the univariable or multivariable model.

Interpretation — Almost all employees returned to work after excision of lumbar disc herniation. Older age, manual job, and prolonged sick leave before the excision of lumbar disc herniation were risk factors for delayed return to work after the surgery.

Return to work (RTW) is an important outcome of lumbar disc herniation surgery, and a key metric for its effectiveness, as it has profound implications for both individual patients and the economy at large. An early RTW is associated with beneficial effects on patients’ physical and mental health and social and economic benefits (Liang et al. 1986, Koenig et al. 2016, Khan et al. 2019).

Favorable outcome of disc herniation surgery when compared with nonoperative treatment was already presented in the 1980s (Weber 1983). This finding has also been recently confirmed in an RCT setting (Bailey et al. 2020). The difference in outcomes between surgically and nonoperatively treated patients may diminish in longer follow-up (Österman et al. 2006). Long duration of leg pain and long preoperative sick leave increase the risk of not returning the work (Kotilainen et al. 1993, Nygaard et al. 2000, Khan et al. 2019). Furthermore, if a worker is on sick leave more than 6 months after the operation, the probability of not returning to work is as high as 50% (Frank et al. 1996). Therefore, the main indication for elective disc herniation surgery is fast relief of the symptoms to enable early RTW and prevent the development of permanent work disability. There are several suggested factors associated with prolonged sick leave such as postoperative leg pain, poor work motivation, and female sex (Graver et al. 1998, Puolakka et al. 2008, Huysmans et al. 2018, Khan et al. 2019). Identifying factors predicting RTW may help in patient selection and setting adequate goals for rehabilitation after the surgery. Previous studies on the subject have had partly contradictory findings.

We therefore assessed health-related risk factors of RTW after lumbar disc herniation surgery, such as factors related to general health, health-risk behaviors, and socioeconomic status in a large cohort of public sector employees. This is a linkage study of national health registers and FPS surveys.

## Patients and methods

### Patients

Patients were identified from the Finnish Public Sector (FPS) study, a nationwide register- and survey-based cohort among employees of 10 municipalities and 6 hospital districts covering a wide range of occupations—from city mayors and doctors to semiskilled cleaners, nurses, and teachers (Airaksinen et al. 2017, Laasik et al. 2019, Lankinen et al. 2019). The cohort members were employed for a minimum of 6 months in the participating organizations between 1991 and 2005 (n = 151,901). Since 1997/1998, repeated questionnaire data at 2- to 4-year intervals has been collected from all employees at work at the time of the survey. Further, the questionnaires were filled out between 1997 and 2005 and the patients underwent surgery between 1999 and 2010. Unfortunately, we do not know the exact time between completing the questionnaire and the surgery.

We derived information on baseline characteristics before the surgery from the closest survey responses, the employers’ records, and national health registers. All participants were linked to data on lumbar disc herniation surgery from the National Hospital Discharge Register, maintained by the National Institute for Health and Welfare, as well as the National Sickness Absence Register, maintained by the Social Insurance Institution of Finland, where all sickness absence periods are medically certified and they are encoded to the register with start and end dates (Laasik et al. 2019, Lankinen et al. 2019). The linkage data were available until December 31, 2011. The ethics committee of the Hospital District of Helsinki and Uusimaa approved the study.

### Type of surgery and patient characteristics

Of the FPS cohort members, 1,706 underwent excision of lumbar disc herniation between 1999 and 2010. Of these, 389 (n = 111 male, n = 278 female; mean age 46y, SD 8.9, range 22–64) had responded to a survey before the surgery and were included in the study. The type of surgery was defined as ABC16 or ABC26 (NOMESCO Classification of Surgical Procedures Version 1.14; the Nordic Medico-Statistical Committee).

### Return to work (RTW)

RTW was determined as how long it took for the participants to return to work, if at all, after the surgery, that is the number of days between the date of discharge and the date of the end of the sick leave. The follow-up time for RTW was 12 months. All Finnish residents aged 16 to 67 years are legitimized to receive daily allowances due to medically certified sickness absence. After a qualifying period of the first 9 days of illness, compensation is paid based on salary for a maximum of 1 year. Overlapping and consecutive periods of sick leave were merged. At 12 months it was dichotomized as yes vs. no, depending on whether or not the patient had returned to work.

### Risk factors for RTW

The participants’ age, sex, and occupational title at the time of the surgery were obtained from the employers’ registers. To measure occupational status, occupational titles, coded according to the International Standard Classification of Occupation (ISCO), were categorized into three groups: higher-grade non-manual workers (e.g., teachers, physicians), lower-grade non-manual workers (e.g., registered nurses, technicians), and manual workers (e.g., cleaners, maintenance workers). Marital status (married or cohabiting vs. single, divorced, or widowed) was obtained from the baseline questionnaire. Age at the time of disc herniation surgery was categorized as age groups < 40 years, 40–50 years, > 50 years.

Information on health and health behaviors was obtained from the baseline questionnaire and national health registers described in detail earlier. Briefly, physical activity was defined as average weekly hours of leisure-time physical activity, categorized into 2 groups, “low” (<1 4 MET/hours/week) and “high” activity (> 14 MET/hours/week) (Kujala et al. 1998). Alcohol consumption was categorized according to the habitual frequencies of drinking beer, wine, and spirits as “none,” “moderate,” and “heavy” consumption. The cut-off for heavy alcohol consumption was set as 210 g/week (Rimm et al. 1999). Smoking status was dichotomized as “currently smoking” vs. “has quit or never smoked.” Self-reported bodyweight and height were used to calculate BMI, which was used to identify obese (BMI ≥ 30) and non-obese (BMI < 30) participants. Psychological distress was measured with the 12-item version of the General Health Questionnaire (GHQ) (Goldberg et al. 1997), using 3/4 positive responses as a cut-off point of psychological distress (“yes” vs. “no”). Participants rated their general health on a 5-point scale (from 1 = “good health” to 5 = “worst health”), and the self-rated health was then dichotomized by categorizing response scores 1 and 2 as good and scores 3 to 5 as poor self-rated health. Data on comorbid chronic conditions—diabetes, coronary heart disease, asthma, chronic obstructive pulmonary disease, and rheumatoid arthritis—was obtained from the Drug Reimbursement Register, which contains information on persons entitled to special reimbursement for treatment of severe chronic illnesses. The presence of comorbidity was then dichotomized as “yes” vs. “no.” Antidepressant and pain medication prescriptions within 100 days of surgery were obtained from Social Insurance Institution of Finland.

### Statistics

The participants were followed from the date of the discharge between January 1, 1999 and December 31, 2010 to the date when an employee returned to work, was granted a disability pension, an old-age pension, died, or end of study, December 31, 2011, whichever came first. A univariable Cox proportional hazards regression model was used for estimation of possible risk factors and hazard ratios with 95% confidence intervals (CI) for RTW (Table). Directed acyclic graph (DAG) analysis on the risk factors for RTW was performed based on the literature and clinical practise to organize variables according to their supposed relation to RTW and to other variables (Figure). For all the variables with potential confounding bias we performed a multivariable analysis (Table). Age, sex, and occupational status were considered as sociodemographic factors in DAG, whereas alcohol consumption, smoking, and physical activity were considered as health-risk behaviors. Further, obesity, self-rated health, psychological distress, comorbidities, antidepressant medication, and pain medication were considered as health in the DAG. All analyses were performed using the SAS statistical software, version 9.1.3 (SAS Institute, Cary, NC, USA).

**Figure.**
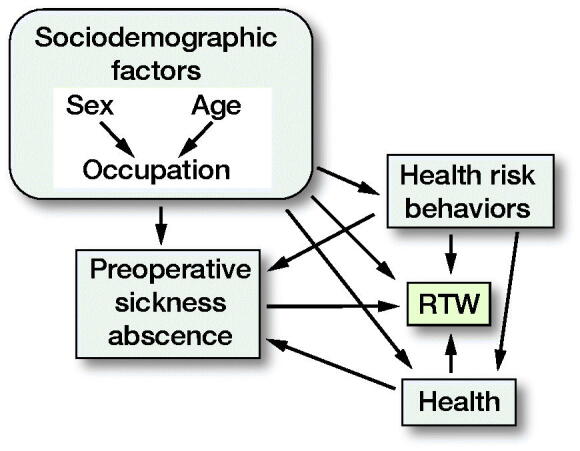
Directed acyclic graph (DAG). Age, sex, and occupational status were considered as sociodemographic factors in DAG, whereas alcohol consumption, smoking, and physical activity were considered as health risk behaviors. Further, obesity, self-rated health, psychological distress, comorbidities, antidepressant medication, and pain medication were considered as health in the DAG. RTW = return to work.

**Table. Ba:** seline characteristics of the patients (n = 389) and their associations with the rate of return to work after excision of lumbar disc herniation. Hazard ratios (HR) and their 95% confidence intervals (CI) are derived from Cox proportional hazard uni- and multivariable analyses

		Separately	Multivariable
		analyzed	model
Factor	n (%)	HR (CI)	HR (CI)
Age (missing n = 0):			
< 40	102 (26)	1.5 (1.1–1.9)	1.4 (1.1–1.9)
40–50	138 (36)	1.3 (1.0–1.7)	1.3 (1.0–1.7)
> 50	149 (38)	reference	reference
Sex (missing n = 0):			
Men	111 (29)	1.1 (0.8–1.3)	1.1 (0.8–1.4)
Women	278 (72)	reference	reference
Married or cohabiting (missing n = 0):			
No	100 (26)	1.2 (0.9–1.5)	1.1 (0.8–1.4)
Yes	289 (74)	reference	reference
Obese (BMI > 30) (missing n = 11):			
No	322 (85)	1.2 (0.9–1.6)	1.2 (0.9–1.7)
Yes	56 (15)	reference	reference
Comorbidities (missing n = 0)			
No	341 (88)	1.2 (0.9–1.7)	1.1 (0.7–1.5)
Yes	48 (12)	reference	reference
Current smoking (missing n = 4):			
No	301 (78)	1.2 (0.9–1.5)	1.0 (0.8–1.4)
Yes	84 (22)	reference	reference
High alcohol consumption (missing n = 2)			
No	349 (90)	1.0 (0.7–1.4)	0.9 (0.6–1.4)
Yes	38 (10)	reference	reference
Occupational status (missing n = 4):			
Non-manual			
Higher level	91 (24)	1.6 (1.2–2.1)	1.6 (1.2–2.1)
Lower level	142 (37)	1.3 (1.1–1.7)	1.3 (1.0–1.7)
Manual	152 (40)	reference	reference
Self-rated health (missing n = 2):			
Good	261 (67)	1.1 (0.9–1.4)	1.0 (0.7–1.2)
Poor	126 (33)	reference	reference
Psychological distress (missing n = 1):			
No	277 (71)	1.1 (0.8–1.3)	1.1 (0.9–1.5)
Yes	111 (29)	reference	reference
Physically active (MET hours >14 h/week) (missing n = 4):			
Yes	295 (77)	1.0 (0.8–1.3)	1.0 (0.8–1.3)
No	90 (23)	reference	reference
Antidepressant medication purchase within 100 days (missing n = 0)			
No	365 (94)	1.1 (0.7–1.7)	1.0 (0.6–1.7)
Yes	24 (6)	reference	reference
Pain medication purchase within 100 days (missing n = 0):			
No	144 (37)	0.9 (0.8–1.1)	1.0 (0.8–1.3)
Yes	245 (63)	reference	reference
Preoperative sickness absence **a** (missing n = 0)			
No	221 (57)	1.3 (1.1–1.6)	1.3 (1.0–1.6)
Yes	168 (43)	reference	reference

a More than 30 days of sickness absence within the 1-year period preceding the operation.

### Ethics, funding, and potential conflicts of interest

Each author certifies that his or her institution has approved the human protocol and that all investigations were conducted in conformity with ethical principles of research. Conflicts of interest and source of funding: None.

## Results

The average age of the patients at the time of surgery was 46 years (SD 8.9, range 22–64) and 72% were women. One third of the patients were in manual work. Almost half had been on sick leave before the operation and 2 out of 3 had used pain medication. Among the patients, 12% had some chronic medical comorbidity, 33% rated their health as poor, and 29% were psychologically distressed. Moreover, 15% were obese, and over 20% were smoking or physically inactive. Purchased prescribed antidepressants were relatively rare (6%) prior to surgery (Table).

### Risk factors for RTW

At 12-month follow-up after the surgery, 371 out of 389 patients (95%) had returned to work after being on sick leave for 78 (SD 77, range 13 to 366) days on average. Occupational status, sickness absence before the surgery, and age were associated with RTW in both a univariable and a multivariable model. Patients with higher level non-manual occupational status had a 1.6 (CI 1.2–2.1) times higher hazard of RTW than manual workers; in patients with ≤ 30 days’ sick leave before the surgery the corresponding hazard was 1.3-fold (CI 1.1–1.6) compared with those with a longer sick leave. Patients aged < 40 years returned to work sooner than patients aged > 50 years (HR 1.5 (CI 1.1–1.9) (Table).

In contrast, socioeconomic factors (sex and marital status), health behaviors (obesity, smoking, physical activity, alcohol consumption, self-related health), chronic medical comorbidities (asthma, diabetes mellitus, rheumatoid arthritis, and coronary artery disease), or psychological distress and purchase of pain or antidepressant medications were not associated with RTW (Table).

## Discussion

This occupational cohort study among 389 public sector employees who underwent lumbar disc herniation surgery showed that 95% of them returned to work within the 12-month follow-up, and on average 78 days (2.5 months) after the surgery. The average sick leave duration is comparable to those published earlier (Huysmans et al. 2018, Than et al. 2016). Younger age, a smaller number of sickness absence days before the surgery, and a higher occupational status were associated with the rate of return to work. In turn, demographic and socioeconomic factors (sex and marital status), health behaviors (smoking, physical inactivity, and excessive alcohol consumption), comorbidities, self-rated poor health, psychological distress, and antidepressant or pain medication prescriptions were not associated with RTW.

Several studies have previously examined predictors of RTW after lumbar discectomy with partly contradictory findings. Than et al. (2016) assessed predictors of RTW at 3 months in 105 patients based on a US neurosurgical registry, and found that younger age was the only statistically significant predictor of postoperative RTW. Sex, BMI, smoking status, and comorbidity were not associated with RTW. In their study, 94% of the patients had returned to work at 12-month follow-up (average 67 days). Also, Paulsen et al. (2020) found in a Danish study of 146 patients that sex, BMI, and smoking status were not associated with RTW. Our results are in accordance with these previous studies, although Than et al. (2016) did not examine occupational status or preoperative sickness absence. Paulsen et al. (2020) stated further that preoperative sick leave was not associated with the duration of postoperative sick leave, whereas occupational status was associated. The latter is in line with our findings, as well as that of Nygaard et al. (2000) who stated that patients with sick leave of more than 7 months before the surgery for lumbar disc herniation surgery were at higher risk of not returning to work.

Contrary to our findings and those of Than et al. (2016) and Paulsen et al. (2020), Sørlie et al. (2012) stated that smoking may be a predictor of prolonged RTW after lumbar microdiscectomy. They assessed 178 Norwegian patients and 25 of the smokers received sickness benefit after 12 months, compared with 19 of the non-smokers. However, their main interest was residual back pain, and potential confounders such as occupational status were not addressed, which may cause bias. Further evidence on the harmful effect of smoking on RTW was presented by Dewing et al. (2008) who claimed that smokers had a lower return to full active military duty after lumbar microdiscectomy (84% had returned to unrestricted duty at 3 years). However, this very young (mean age 27 years) and active population differs basically from that of ours (mean age 46 years, public sector workers), which may explain the difference.

Schade et al. (1999) studied 46 patients undergoing lumbar discectomy with pain relief, reduction of disability in daily activities, and RTW as outcomes at 2 years. MRI-identified nerve root compromise and social support from the spouse were independent predictors of pain relief, whereas RTW at 2 years after surgery was best predicted by preoperative depression and work stress. They stated that the most important finding of the study was that RTW was not influenced by any clinical findings or MR-identified morphological alterations, but solely by psychological factors (i.e., depression) and psychological aspects of work (i.e., work stress). Our results are not consistent with these findings. In our study, psychological distress or antidepressant medication were not associated with RTW. It should be noted that compared with our study, their sample size was remarkably smaller (46 vs. 389 patients), which may have influenced results and caused bias due to chance findings or selection of patients.

Vucetic et al. (1999) found in 156 patients that factors predicting RTW at 2 years after surgery for lumbar disc herniation were: no preoperative comorbidity, duration of sciatica less than 7 months, higher education, age younger than 41 years, male sex, and no previous non-spinal surgery. In our study, we did not have data on duration of symptoms, but we considered the length of the preoperative sickness absence as a proxy. Further, we measured socioeconomic status from occupational status and not from education. Longer time on preoperative sick leave was also a predictor for decreased working capacity after lumbar disc herniation surgery in the Swedish population (Silverplats et al. 2010). Also, Schoeggl et al. (2003) found that patients with strenuous occupations had a decreased RTW compared with patients with less strenuous or sedentary occupations. Sex was not associated with RTW in our study, nor in the study of Than et al. (2016) or Paulsen et al. (2020). Female sex was associated with not returning to work in the study of Jensdottir et al. (2007), but their patients had been operated on in the early 1980s in Iceland, which may explain the finding. Comorbidity was not an issue based on our data, contrary to that of Vucetic et al. (1999). Our study patients were solely public sector workers (no farmers, construction workers, or soldiers), which may reduce the generalizability of the findings to other populations.

Our results need to be interpreted with some strengths and limitations in mind. Compared with previous studies, our cohort size was the second largest we are aware of after that of Schoeggl et al. (2003). The studied sample came from a well-characterized occupational cohort and represented a wide range of occupations. Comprehensive data had been gathered before the surgery on health and health-risk behaviors. All data was linked to reliable national health registers including detailed information regarding the date of the operation and the beginning and end dates of all periods of sickness absence, enabling accurate estimation of the timing of return to work. Many predictors of RTW, such as occupational status, sickness absences, antidepressant and pain medications before the operation, and comorbid medical conditions, were measured objectively from the registers.

As a limitation, the generalizability of our findings may be affected by the differences in welfare, pension, and workers’ compensation schemes in different countries (Scott et al. 2017). The cohort studied was limited to employees in the public sector predominated by women in a Nordic welfare state. We were not able to address changes in compensation and economic conditions in Finland. However, the terms of employment in the public sector were relatively stable during the time of the study, offering high job security to the employees and no major changes in sickness absence compensation. No data on workplace adjustments before or after the surgery was available. We did not have data on surgical techniques, so we were not able to assess whether less invasive surgical techniques were found to result in increased RTW compared with more invasive techniques. No data on radiographic imaging, complications, or repeated surgery was available. Another limitation is that we were not able to assess patient satisfaction reports or functional outcome scores. RTW may also be influenced by patients’ interactions with healthcare professionals as well as patients’ pre-surgery expectations (Bardgett et al. 2016). One limitation of our study was also that we were not able to separate those who had quit smoking from those who have never smoked.

## Conclusion

In this occupational cohort of 389 employees, 95% of the participants had returned to work at 12 months after excision of lumbar disc herniation. Older patients in manual work with prolonged sick leave before the excision of lumbar disc herniation were at increased risk of a slower return to work after the surgery.
